# Early identification of the efficacy of 0.125% atropine treatment for children with Myopia: A prospective pilot study

**DOI:** 10.1371/journal.pone.0327354

**Published:** 2025-08-07

**Authors:** Zi-Rong Chen, Tsung-Yao Wan, Lan-Hsin Chuang, Chi-Chun Lai, Yih-Shiou Hwang, Yu-Kai Kuo, Ho-Min Chen, Po-Chun Chang, Hung-Chi Chen, Chun-Fu Liu

**Affiliations:** 1 Department of Family Medicine, Cheng Ching Hospital, Taichung, Taiwan; 2 College of Medicine, Chang Gung University, Taoyuan, Taiwan; 3 Department of Education, China Medical University Hospital, China Medical University, Taichung, Taiwan; 4 Department of Sports Medicine, China Medical University, Taichung, Taiwan; 5 Department of Ophthalmology, Chang Gung Memorial Hospital, Keelung, Taiwan; 6 Department of Ophthalmology, Chang Gung Memorial Hospital, Linkou, Taiwan; 7 Department of Ophthalmology, Jen-Ai Hospital Dali Branch, Taichung, Taiwan; 8 Department of Ophthalmology, Chang Gung Memorial Hospital, Xiamen Branch, Xiamen, China; 9 Center for Tissue Engineering, Chang Memorial Hospital, Linkou, Taiwan; 10 Program in Molecular Medicine, National Yang Ming University, Taipei, Taiwan; University of Missouri-Columbia, UNITED STATES OF AMERICA

## Abstract

**Purpose:**

This study aimed to investigate whether early axial length (AL) changes in the short term after 0.125% atropine treatment could predict long-term axial elongation in children with myopia.

**Methods:**

This was a prospective cohort study involving children aged 5–15 years with myopia who were treated with 0.125% atropine for myopia control. AL was measured 1–2 months after starting treatment and then every 3 months for follow-up visits. Regression analysis was used to develop a model of AL changes with time. A generalized estimating equation (GEE) model was then used to identify correlations between the early AL changes and long-term AL changes.

**Results:**

Eighty eyes of 40 patients (mean age 8.4 years) were included in the final analysis. The estimation curve of AL changes with time indicated that the AL decreased at 67 days (the turning point in the regression model) after 0.125% atropine treatment and then increased gradually with time. Univariate GEE showed that a larger AL elongation in the initial 4 months was significantly associated with AL changes at 6 months (β = 0.354, *P* = 0.020, 6 ~ 12 months period from baseline) and 12 months (β = 0.560, *P* = 0.045, 6 ~ 18 months period from baseline) after that period in all myopic eyes.

**Conclusions:**

The magnitude of AL elongation in the initial 4 months of 0.125% atropine treatment correlated positively with the further half-year and one-year AL changes. Identifying these changes may be useful for controlling refractory myopia in children.

## Introduction

Myopia is a refractive disorder that leads to impairments in distance vision and has a considerable prevalence in modern society. The prevalence of myopia is increasing worldwide, and the global prevalence was 22.9% in 2000 and is estimated to increase to 49.8% in 2050 [1]. In Taiwan from 1983 to 2000, the prevalence of myopia increased from 5.8% to 21.0% among 7-year-old children and from 74% to 84% among 18-year-olds [2]. Pathological myopia is defined as excessive axial myopia that leads to structural changes in the posterior segment of the eye, which can lead to irreversible visual impairment and impaired quality of life [3,4]. Axial myopia is a progressive disease that occurs in school-age children and results from excessive axial length (AL) elongation [4]. Therefore, early and continuous control of axial myopia is essential for preventing visual impairment. Among the current strategies for controlling myopia, the pharmacological method is used most often because of supportive evidence, better therapeutic range, and cost-effectiveness in Taiwan. Lower-dose atropine has fewer adverse and rebound effects in patients [5–7]. However, in Taiwan, the National Health Insurance (NHI) covered 0.125% atropine for myopia control a long time ago and remains the most frequently prescribed in routine practice due to its balance of efficacy and accessibility. Fortunately, it has recently started to cover 0.01% [8,9].

Previous studies and clinical experience suggest that some people may respond poorly to lower-dose atropine therapy. In the ATOM 2 study, 4.3%, 6.4%, and 9.3% of children given atropine at a dose of 0.5%, 0.1%, or 0.01%, respectively, had myopia progression ≥−1.5 diopters (D) over the initial 2 years of active treatment [5]. At present, there is no useful early indicator for predicting the responsiveness of patients to atropine treatment. It is important to identify patients with poor responsiveness to lower-concentration atropine therapy alone as early as possible because there are combination therapies with other optical methods to control myopia or enhanced control strength with increasing atropine concentration [6,10–13].

Current evidence indicates that the AL of eyes under atropine treatment decreases for a period of time and then increases gradually [5,7,14]. However, no studies have examined the relationships between the fluctuation in AL and long-term AL elongation periods after atropine treatment. To explore these possible associations, we conducted a prospective longitudinal study of school-aged children who received 0.125% atropine treatment in Taiwan. We examined the relationships between the AL shortening period, initial AL elongation period, and long-term AL changes.

## Methods

### Patients

**Fig 1:**
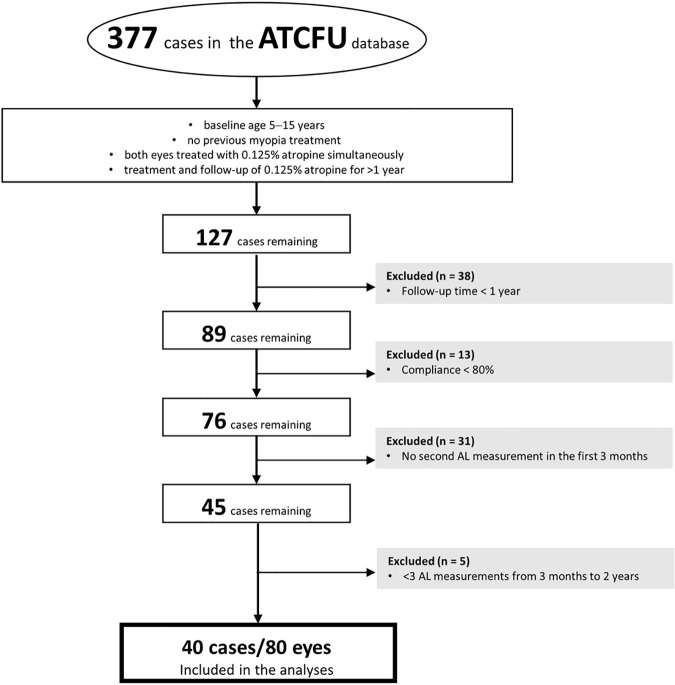
Flow diagram of the patient selection into the final analysis.

This prospective cohort study included patients who were followed up regularly for myopia control at Chang Gung Memorial Hospital, Keelung, Taiwan. This study protocol was approved by the Institutional Review Board of Chang Gung Memorial Hospital (Approval Number: 202000452B0C602) and followed the tenets of the Declaration of Helsinki. The consent form was written by both the patients and their parents after a detailed explanation of the study. The recruitment period for this study started from 2021/01/11, and the study completed, including all data collection, follow up and analysis, was until 2023/06/30. The inclusion and exclusion criteria for this study are summarized in Fig 1. Ultimately, 80 eyes of 40 children were included in the final analysis.

### Standard myopia control strategy

All patients who provided informed consent were monitored with a standard myopia control strategy. For myopia control in children, we recorded their baseline cycloplegic refractive error and AL at their first visit and then planned to follow up every 3–4 months. For children whose AL elongated rapidly (annual increase of AL more than 0.3 mm before age 12 or 0.2 mm after age 12) or indicated a high risk of progression to high myopia (>6 D) (calculate AL growth rate and linear estimate that AL may exceed 26 mm when age 18), we started myopia control treatment at the second follow-up visit with subsequent scheduled visits at 1, 3, 6, 9, and 12 months, and every 3–6 months thereafter. The children who did not meet the criteria for rapid progression and high risk were considered to have slow elongation and low risk, and were observed every six months until their AL changes had accelerated to the range requiring treatment.

Because compliance, treatment with other options for myopia treatment, such as a change in atropine concentration, orthokeratology [15], or myopia control spectacles [16,17], can affect myopia and AL, patients with poor compliance (compliance < 80%) or those receiving other treatments were excluded from the study.

### Ophthalmic examinations

Ocular examinations included uncorrected distance visual acuity and best-corrected visual acuity measurements, slit-lamp anterior segment examination, and measurements of objective refraction errors before and after cycloplegia using an Auto Ref/Keratometer (ARK-1a/ARK-1; Nidek Co., Ltd., Gamagori, Aichi, Japan) performed at the first visit. The cycloplegic refractive error was obtained 1 h after the first instillation of 1% tropicamide (Mydriacyl; Alcon Vision, LLC, Fort Worth, TX, USA) plus 10% phenylephrine hydrochloride (Phenylephrine Eye Drops; Wu Fu Laboratories Co. Ltd., Yilan, Taiwan). For cycloplegia induction, both drops were instilled together three times at 10 min intervals. AL, keratometry (Km), and anterior chamber depth (ACD) were measured with an optical AL measuring device (IOLMaster 500, Carl Zeiss Meditec AG, Jena, Germany).

### Statistical analysis

**Fig 2:**
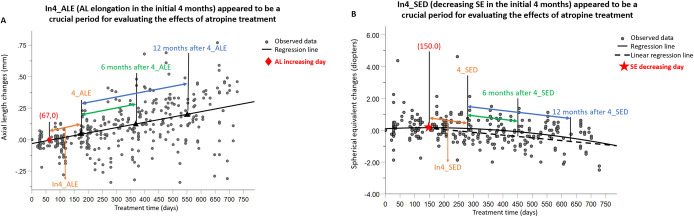
Changes in AL (A) and SE (B) according to the treatment time estimation curve in patients given 0.125% atropine. **(A)** The crucial period and outcome measurement period in the current study. A larger In4_ALE correlated significantly with larger changes in AL at 6 months after In4_ALE in all myopic children. Associated data are presented in Table 2. **(B)** The crucial period and outcome measurement period in the current study. A larger In4_SED correlated significantly with larger changes in SE at 6 months after In4_SED. Associated data are presented in Table 3. In4_ALE, AL elongation in the initial 4 months; In4_SED, decreasing SE in the initial 4 months; 4_ALE, the day at 4 months after AL elongation; 4_SED, the day at 4 months after SE decreasing. AL, axial length; SE, spherical equivalent.

The data were analyzed using IBM SPSS Statistics for Windows (version 23.0; IBM Corp., Armonk, NY, USA). Regression analysis was used to identify significant relationships between treatment time (days) and the changes in AL (mm) and spherical equivalent (SE). We created a regression estimation curve to note the “AL increasing day” and the “SE decreasing day” (Fig 2).

**Table 1:** Patients’ demographics and clinical parameters.

	Number of eyes (*n* = 80)
Baseline age (years)	8.4 ± 2.12
Boy eyes	44 (55%)
Baseline AL (mm), *n* = 78	23.99 ± 1.00
Baseline Km (D), *n* = 42	43.12 ± 1.43
Baseline ACD (mm), *n* = 28	3.61 ± 0.25
Baseline SE (D), *n* = 72	–1.77 ± 1.78
Baseline sphere (D), *n* = 72	–1.14 ± 1.70
Baseline astigmatism amount (D), *n* = 72	1.30 ± 1.22
Follow-up time (months)	21.3 ± 8.4

Continuous data are presented as mean ± standard deviation. *n*, number of eyes; AL, axial length; Km, keratometry; ACD, anterior chamber depth; SE, spherical equivalent refractive error.

The total follow-up period in months was calculated as (the number of days between the last and first AL measurement)/30.44.

**Table 2:** Associations between demographic variables, In4_ALE and AL changes at 6 and 12 months after 4_ALE (**n* *= 80).

Dependent variable: AL changes at 6 months after 4_ALE						
	Univariate			Multivariable		
Variable	Estimate	(95% CI)	*P* value	Estimate	(95% CI)	*P* value
Baseline age (years)	–0.020	(–0.028, –0.012)	**<0.001** ^*^	–0.018	(–0.026, –0.009)	**<0.001** ^*^
Male sex	–0.046	(–0.088, –0.004)	**0.032** ^*^	–0.019	(–0.057, 0.019)	0.332
Baseline AL (mm), *n* = 78	–0.020	(–0.044, 0.003)	0.093			
Baseline SE (D), *n* = 72	0.005	(–0.011, 0.020)	0.557			
Baseline sphere (D), *n* = 72	0.008	(–0.006, 0.022)	0.245			
Baseline astigmatism amount (D), *n* = 72	0.013	(–0.005, 0.031)	0.167			
Follow-up time (months)	<0.001	(–0.003, 0.004)	0.833			
In4_ALE	0.354	(0.056, 0.652)	**0.020** ^*^	0.051	(–0.236, 0.338)	0.728
Dependent variable: AL changes at 12 months after 4_ALE						
	Univariate			Multivariable		
Variable	Estimate	(95% CI)	*P* value	Estimate	(95% CI)	*P* value
Baseline age (years)	–0.039	(–0.053, –0.025)	**<0.001** ^*^	–0.034	(–0.047, –0.021)	**<0.001** ^*^
Male sex	–0.055	(–0.137, 0.027)	0.188			
Baseline AL (mm), *n* = 78	–0.028	(–0.071, 0.016)	0.210			
Baseline SE (D), *n* = 72	–0.005	(–0.038, 0.028)	0.759			
Baseline sphere (D), *n* = 72	0.005	(–0.025, 0.035)	0.745			
Baseline astigmatism amount (D), *n* = 72	0.041	(0.005, 0.077)	**0.025** ^*^	0.031	(–0.003, 0.066)	0.078
Follow-up time (months)	–0.002	(–0.009, 0.004)	0.488			
In4_ALE	0.560	(0.012, 1.108)	0.045*	–0.092	(–0.627, 0.443)	0.735

* P < 0.05, generalized estimating equation.

AL, axial length; SE, spherical equivalent refractive error; CI, confidence interval; In4_ALE, AL elongation in the initial 4 months; 4_ALE, the day at 4 months after AL elongation.

**Table 3:** Associations between demographic variables, In4_SED and SE changes at 6 and 12 months after 4_SED (*n* = 80).

Dependent variable: SE changes at 6 months after 4_SED						
	Univariate			Multivariable		
Variable	Estimate	(95% CI)	*P* value	Estimate	(95% CI)	*P* value
Baseline age (years)	0.061	(0.019, 0.104)	**0.005** ^*^	0.052	(0.021, 0.084)	**0.001** ^*^
Male sex	0.139	(–0.096, 0.375)	0.246			
Baseline AL (mm), *n* = 78	0.053	(–0.068, 0.175)	0.389			
Baseline SE (D), *n* = 72	–0.020	(–0.085, 0.044)	0.539			
Baseline sphere (D), *n* = 72	–0.030	(–0.093, 0.033)	0.355			
Baseline astigmatism amount (D), *n* = 72	–0.027	(–0.127, 0.073)	0.598			
Follow-up time (months)	0.002	(–0.016, 0.019)	0.858			
In4_SED	0.666	(0.101, 1.230)	**0.021** ^*^	0.606	(0.087, 1.125)	**0.022** ^*^
Dependent variable: SE changes at 12 months after 4_SED						
	Univariate			Multivariable		
Variable	Estimate	(95% CI)	*P* value	Estimate	(95% CI)	*P* value
Baseline age (years)	0.135	(0.053, 0.218)	**0.001** ^*^			
Male sex	0.338	(–0.122, 0.798)	0.150			
Baseline AL (mm), *n* = 78	0.074	(–0.164, 0.312)	0.544			
Baseline SE (D), *n* = 72	–0.024	(–0.171, 0.124)	0.753			
Baseline sphere (D), *n* = 72	–0.038	(–0.189, 0.113)	0.621			
Baseline astigmatism amount (D), *n* = 72	–0.037	(–0.236, 0.161)	0.713			
Follow-up time (months)	0.006	(–0.027, 0.039)	0.719			
In4_SED	1.147	(–0.107, 2.402)	0.073			

* P < 0.05, generalized estimating equation.

AL, axial length; SE, spherical equivalent refractive error; CI, confidence interval; In4_SED, decreasing SE in the initial 4 months; 4_SED, the day at 4 months after SE decreasing.

Univariate generalized estimating equations (GEEs) were used to identify correlations between AL elongation in the initial 4 months (In4_ALE) and AL changes at 6 and 12 months after In4_ALE. The SE was also analyzed in a similar way to identify correlations between the decreasing SE in the initial 4 months (In4_SED) and changes in the SE at 6 and 12 months after In4_SED. Univariate GEEs were also used to identify significant correlations between demographic variables and the changes in the AL and SE at 6 and 12 months after In4_ALE and In4_SED, respectively (Tables 2 and 3).

Significant variables in the univariate GEEs were included in a multivariable GEE. Independent working correlation structure and robust standard errors were adopted to identify the significance of parameters with the lowest corrected quasi-likelihood under the independence model criterion (QICC). Two-tailed *P* values < 0.05 were accepted as significant, and no adjustment of alpha error was made in this study.

Since both eyes from each subject were included, GEEs with robust standard errors and independent working correlation structures were used to account for intra-subject correlation and prevent overestimation of statistical significance.

Given that every patient’s follow-up date deviated somewhat because of personal reasons, when we detected the AL increasing day in the regression model, we used the interpolation method between the two closest follow-up days to estimate the AL on the AL increasing day and the day at 4 months after AL elongation. A similar method was used for the SE.

## Results

### Demographic data for the enrolled patients

A flow chart for patient enrollment is presented in Fig 1. A total of 377 patients were enrolled. We excluded the cases that mismatched the criteria for this study. Ultimately, 80 eyes for 40 patients were included in the analysis.

The demographic data and clinical characteristics are shown in Table 1. The average age was 8.4 years. The average baseline AL was 23.99 mm, and the baseline SE was –1.77 D. The mean follow-up time was 21.3 months. Because some data were lost at the first visit, ocular examination data such as baseline AL, Km, ACD, and SE, are labeled by case numbers.

### AL decreased in the first 67 days and then increased gradually; SE increased in the first 150 days and then decreased gradually

The results of the regression analysis between treatment time (days) and AL changes (mm) are shown in Fig 2A. The estimation curves showed an average “AL increasing day” at day 67 and an average “SE decreasing day” at day 150 (Fig 2B). The ACD and Km did not change significantly with treatment time (S1 and S2 Figs).

### AL changes at 6 and 12 months after AL elongation in the initial 4 months (6 ~ 12 and 6 ~ 18 months periods from baseline), Correlated positively with AL elongation in the initial 4 months (In4_ALE) in the children with myopia

In the univariate GEEs, younger age at the baseline (β = –0.020, *P* < 0.001) and larger In4_ALE (β = 0.354, *P* = 0.020) correlated significantly with larger AL changes at 6 months after In4_ALE in all myopic eyes. This finding suggests that the In4_ALE was a crucial period for evaluating the effects of atropine treatment in these children. Male sex (β = –0.046, *P* = 0.032) also correlated significantly with smaller AL changes at 6 months after In4_ALE. In the multivariable GEE that included these three variables, only the baseline age (β = –0.018, *P* < 0.001) remained significantly associated with the AL changes at 6 months after In4_ALE.

In the univariate GEE, younger age at the baseline (β = –0.039, *P* < 0.001), greater baseline astigmatism (β = 0.041, P = 0.025), and larger In4_ALE (β = 0.560, P = 0.045) correlated significantly with the AL changes at 12 months after In4_ALE. In the multivariable GEE, only a younger baseline age (β = –0.034, *P* < 0.001) remained significantly correlated with the AL changes at 12 months after In4_ALE. These results are summarized in Table 2 and Fig 2A.

### SE changes at 6 and 12 months after In4_SED Correlated positively with In4_SED in all myopic children

In the univariate GEEs, a younger baseline age (β = 0.061, *P* = 0.005) and larger In4_SED (β = 0.666, P = 0.021) correlated significantly with the SE changes at 6 months after In4_SED in all myopic eyes. In the multivariate GEE, a younger baseline age (β = 0.052, P = 0.001) and larger In4_SED (β = 0.606, P = 0.022) remained significantly correlated with SE changes 6 months after In4_SED.

In the univariate GEE, only a younger baseline age (β = 0.135, *P* = 0.001) correlated significantly with SE changes at 12 months after In4_SED. These results are summarized in Table 3 and Fig 2B.

## Discussion

To our knowledge, this is the first study to evaluate the long-term control of myopia by measuring short-term AL elongation after initiating 0.125% atropine therapy in children with myopia. We found that the AL changes at 6 and 12 months after In4_ALE correlated significantly with In4_ALE. This association may be useful for monitoring the long-term responsiveness to topical atropine within 6 months of treatment in clinical practice.

Previous studies have reported that a proportion of children with myopia respond poorly to atropine therapy; the risk factors for a poor response include a younger age and a family history of myopia [18–20]. In our study, a younger baseline age was associated with larger changes in AL at 6 and 12 months after In4_ALE and with larger changes in SE at 6 and 12 months after In4_SED (Tables 2 and 3), consistent with the results of the LAMP study [21–24]. These findings suggest that faster myopia progression rates are expected in younger patients. Therefore, the earlier the myopia onset, the greater the attention needed; that is, early evaluation of In4_ALE or In4_SED is crucial for identifying poor responders to atropine therapy.

While larger In4_ALE was significantly associated with greater AL elongation at both 6 and 12 months in univariate analyses, these associations did not remain statistically significant after adjusting for age in the multivariable GEE models. This suggests that age may play a more dominant role in determining long-term axial progression, potentially moderating the predictive value of early AL changes. However, as a pilot study, the result needs further larger study to prove. These findings highlight the importance of age-adjusted models for further study design and interpreting early treatment response [23,24].

Atropine has a dose-dependent effect. According to the LAMP study, 0.05% atropine was more effective than 0.01% and 0.025% for control of myopia in the first year. Moreover, the results of its further study showed that 0.05% atropine is the optimal concentration for 2 years [21,22]. In the ATOM 2 study, the mean myopia progression at 2 years was –0.30 ± 0.60, –0.38 ± 0.60, and –0.49 ± 0.63 D in the 0.5%, 0.1%, and 0.01% atropine groups, respectively [5]. These findings suggest that patients likely to be poor responders to 0.125% atropine, the concentration of atropine should be increased earlier to provide better control of myopia. In our study, the In4_ALE correlated positively with long-term AL changes. Our findings suggest that months 2–6 after atropine treatment represents a crucial period for determining the response to atropine treatment. However, each patient’s individual regression curve for In4_ALE may be more useful for evaluating long-term control effect after further studies.

As shown in the regression curves for the AL and SE in the atropine-treated eyes, the AL shortened for up to 67 days at the beginning of treatment and then elongated gradually. After the first 67 days, the curve tended to become linear. These findings suggest that long-term AL changes can be estimated by observing the AL changes during the initial elongation period. The early recognition of poor responders may increase the treatment’s effectiveness by allowing the change in control strategy or introduction of other strategies earlier [7].

In our regression analysis, the turning point for AL was observed on day 67, while the SE continued to increase until approximately day 150. This 83-day gap may reflect a lag between structural changes in the eye and their optical manifestation. While AL elongation directly contributes to myopia progression, SE is influenced not only by AL but also by accommodation status, corneal power, and lens characteristics [25]. Especially, the accommodative ability may not be fully paralyzed by atropine 0.125% treatment in a short time. These may affect the SE and delay its stabilization [5]. Additionally, changes in choroidal thickness or posterior segment remodeling may lead to an earlier reversal in AL and SE [26,27]. This discrepancy suggests that reliance on SE alone for evaluating early treatment efficacy may delay the timing for effect evaluation and reinforce the utility of AL as a primary metric in monitoring myopia control interventions under atropine 0.125% treatment [28].

The initial period of atropine treatment caused AL shortening. One possible explanation is that, during accommodation, biomechanical forces cause the anterior and inward displacement of the ciliary body. This phenomenon is thought to produce generalized choroidal thinning and result in axial elongation. Greater choroidal thinning during accommodation has been reported in myopes compared with emmetropes, which suggests that the myopic choroid and AL may have a greater propensity to thin and elongate, respectively [7,18,26]. Atropine is used to paralyze the ciliary muscles to alter the accommodation process via the antimuscarinic effect, which could cause a hyperopic shift in atropine-treated eyes. Other researchers have suggested that the alterations in mechanical forces associated with paralysis of the ciliary muscles may cause the young elastic eyeball to change from a prolate to a more oblate shape or a change in the choroidal or scleral thickness, which could lead to an overall reduction in AL. Changes in biometric parameters have also been explored [25,27–30].

We found that AL was shortened within the first 67 days, after which it elongated progressively and linearly. Measuring only AL during this shortening period may have led to an overestimation of the treatment effects of atropine in other studies [5,21,22,31]. We also found that the short-term AL changes (β = 0.732, *P* = 0.004) were significantly associated with the annual AL changes after adjusting for age and sex (S1 Table). Future studies should exclude data obtained during the AL fluctuation period of around 2 months to prevent overestimation of the effects of atropine treatment.

A positive correlation between the female sex and a faster myopia progression rate was also observed in our study (Table 2). Female sex was a significant predictor of myopic progression (change in AL) at 6 months after In4_ALE (β = –0.046, *P* = 0.032). By contrast, the female sex was not a significant predictor of SE changes at 6 and 12 months after In4_SED and of AL changes at 12 months after In4_ALE. Since AL elongation can be influenced by environmental and genetic factors, although the associations are yet to be determined [4,32]. Changes in AL may also be associated with lower participation in outdoor activities among school-aged girls [33]. Furthermore, an earlier study suggested that AL changes should correlate strongly with SE changes [34], but a small sample size or selection bias may also have contributed to this bias. Further cohort studies are needed to clarify whether sex plays a role in the development of myopia.

Our study has several limitations, including its small sample size, loss of some Km and ACD data, and lack of detailed genetic and environmental information. However, we used the changes in AL and SE as the main outcome measures, and we acquired these data by direct measurement with the same machine for each patient, which may have minimized the risk of bias. To strengthen future research, larger-scale, multicenter prospective studies are needed to validate our findings. Such studies could also help clarify age-specific treatment responses, determine optimal monitoring intervals, and refine early predictive indicators of poor response to atropine therapy.

In conclusion, our study found that a larger AL elongation in the initial 4 months (In4_ALE) after starting 0.125% atropine treatment was associated with larger long-term AL elongation. This finding may be useful for identifying poor responders to atropine treatment in a short time. Future studies should be cautious about overestimating the effects of atropine if including the data for the AL shortening period. Larger-scale prospective studies are needed.

## Supporting information

S1 Table Associations between annual AL/SE changes and all demographics data (n = 80). Dependent variable: annual AL changes (mm), annual SE changes (diopter). Annual AL changes (mm) were calculated as [(the difference between the last and the first AL, mm)/(the number of days between the last and the first AL measurement)]×365.25. Annual SE changes (diopter) were calculated as [(the difference between the last and the first SE, diopter)/(the number of days between the last and the first SE measurement)]×365.25. *P < 0.05, generalized estimating equation. AL, axial length; SE, spherical equivalent refractive error; CI, confidence interval.
(DOCX)

S1 Fig The estimation curve for changes in the anterior chamber depth (ACD) and treatment time after 0.125% atropine treatment. Changes in ACD were not significant with treatment time.
(DOCX)

S2 Fig The estimation curve for changes in the mean corneal power (Km) and treatment time after 0.125% atropine treatment. Changes in Km were not significant with treatment time.
(DOCX)
