# Supplementary material for: Early identification of the efficacy of 0.125% atropine treatment for children with Myopia: A prospective pilot study
Source: PLoS One. 2025 Aug 7;20(8):e0327354. doi: 10.1371/journal.pone.0327354 (PMC12331118; doi:10.1371/journal.pone.0327354)
Supplement: S1 Table — Dependent variable: annual AL changes (mm), annual SE changes (diopter). Annual AL changes (mm) were calculated as [(the difference between the last and the first AL, mm)/(the number of days between the last and the first AL measurement)]×365.25. Annual SE changes (diopter) were calculated as [(the difference between the last and the first SE, diopter)/(the number of days between the last and the first SE measurement)]×365.25. *P < 0.05, generalized estimating equation. AL, axial length; SE, spherical equivalent refractive error; CI, confidence interval. (DOCX) [file pone.0327354.s001.docx]

Supporting Table S1. Associations between annual AL/SE changes and all demographics data (n=80)

| Dependent variable: annual AL changes (mm) | | | | | | |
| --- | --- | --- | --- | --- | --- | --- |
|  | Univariate | | | Multivariable | | |
| Variables | Estimate | (95% CI) | P value | Estimate | (95% CI) | P value |
| Baseline age (years) | -0.042 | (-0.053, -0.031) | **<0.001*** | -0.036 | (-0.045, -0.026) | **<0.001*** |
| Male sex | -0.087 | (-0.165, -0.009) | **0.029*** | 0.471 | (0.383, 0.560) | **<0.001*** |
| Baseline AL (mm), n=78 | -0.026 | (-0.072, 0.019) | 0.258 |  |  |  |
| Baseline SE (diopter), n=72 | 0.006 | (-0.025, 0.037) | 0.695 |  |  |  |
| Baseline sphere (diopter), n=72 | 0.014 | (-0.016, 0.045) | 0.352 |  |  |  |
| Baseline astigmatism amount (diopter), n=72 | 0.029 | (-0.002, 0.060) | 0.069 |  |  |  |
| Follow up time (months) | -0.001 | (-0.006, 0.005) | 0.827 |  |  |  |
| Short term AL changes (67 days) | 0.998 | (0.321, 1.675) | **0.004*** | 0.732 | (0.232, 1.231) | **0.004*** |
| Dependent variable: annual SE changes (diopter) | | | | | | |
|  | Univariate | | | Multivariable | | |
| Variables | Estimate | (95% CI) | P value | Estimate | (95% CI) | P value |
| Baseline age (years) | 0.038 | (-0.011, 0.088) | 0.128 |  |  |  |
| Male sex | 0.203 | (-0.248, 0.654) | 0.378 |  |  |  |
| Baseline AL (mm), n=78 | -0.028 | (-0.225, 0.168) | 0.777 |  |  |  |
| Baseline SE (diopter), n=72 | -0.124 | (-0.377, 0.130) | 0.340 |  |  |  |
| Baseline sphere (diopter), n=72 | -0.117 | (-0.318, 0.084) | 0.254 |  |  |  |
| Baseline astigmatism amount (diopter), n=72 | 0.063 | (-0.316, 0.442) | 0.745 |  |  |  |
| Follow up time (months) | -0.027 | (-0.064, 0.010) | 0.147 |  |  |  |
| Short term SE changes (150 days) | 0.947 | (0.429, 1.466) | **<0.001*** |  |  |  |

Dependent variable: annual AL changes (mm), annual SE changes (diopter).

Annual AL changes (mm) were calculated as [(the difference between the last and the first AL, mm)/(the number of days between the last and the first AL measurement)]×365.25.

Annual SE changes (diopter) were calculated as [(the difference between the last and the first SE, diopter)/(the number of days between the last and the first SE measurement)]×365.25.

*P < 0.05, generalized estimating equation.

AL, axial length; SE, spherical equivalent refractive error; CI, confidence interval.
