# Supplementary material for: Early identification of the efficacy of 0.125% atropine treatment for children with Myopia: A prospective pilot study
Source: PLoS One. 2025 Aug 7;20(8):e0327354. doi: 10.1371/journal.pone.0327354 (PMC12331118; doi:10.1371/journal.pone.0327354)
Supplement: S1 Fig — Changes in ACD were not significant with treatment time. (DOCX) [file pone.0327354.s002.docx]

**Supporting Figure S1**


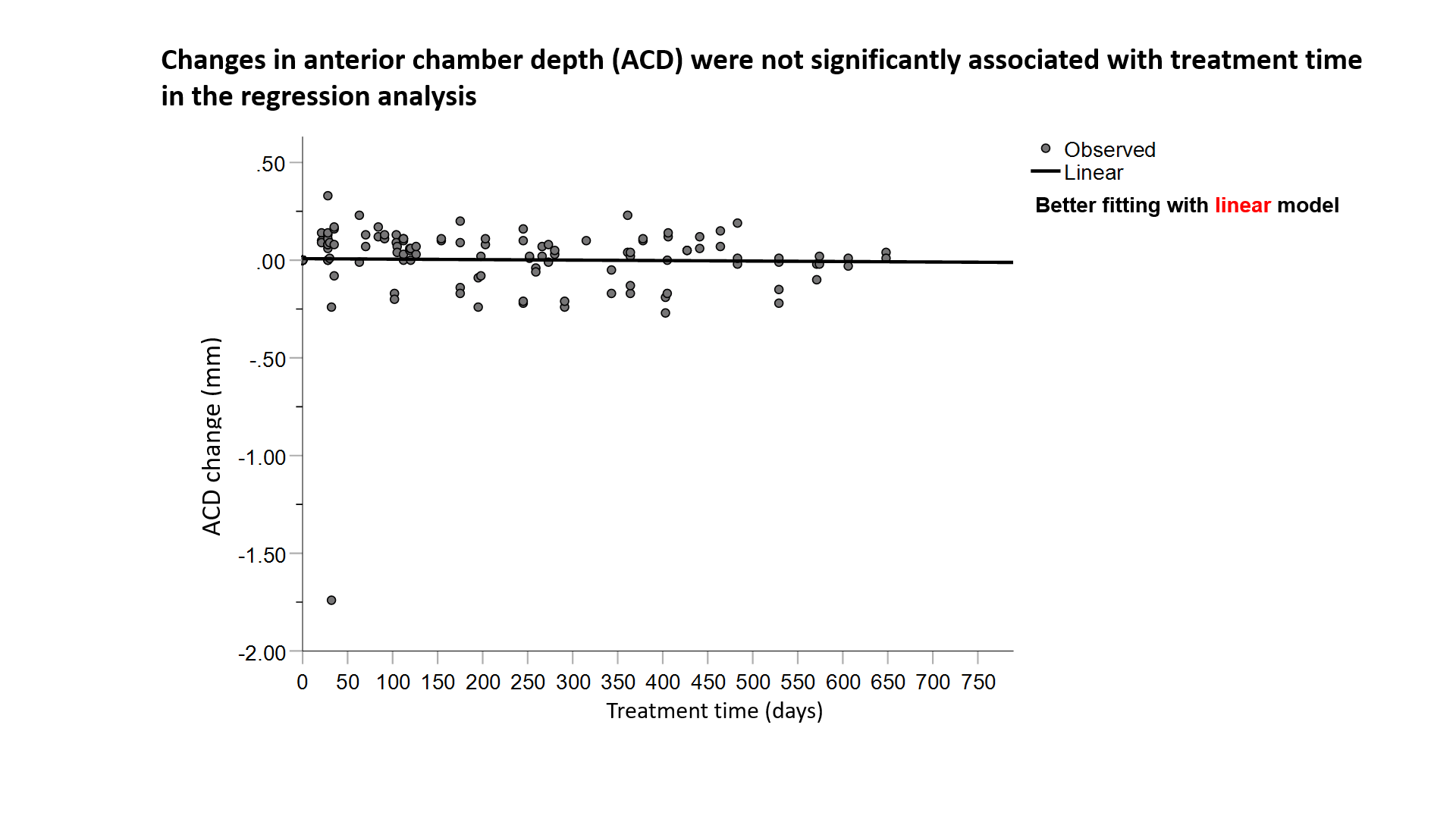


The estimation curve for changes in the anterior chamber depth (ACD) and treatment time after 0.125% atropine treatment. Changes in ACD were not significant with treatment time.
